# Back to the future: Alzheimer's disease heterogeneity revisited

**DOI:** 10.1016/j.dadm.2015.05.006

**Published:** 2015-06-28

**Authors:** Rhoda Au, Ryan J. Piers, Lee Lancashire

**Affiliations:** a Department of Neurology and Framingham Heart Study, Boston University School of Medicine, Boston, MA, USA; b IP & Science, Thomson Reuters, London, UK

The financial burden of caring for 5.2 million people with Alzheimer's disease (AD) is $214 billion. This number will rise to $1.2 trillion by 2050 if the projected tripling to 16 million cases does occur. Importantly, these estimates are likely to be conservative, given the numerous causes of dementia, underscoring the imperative for treatment. Furthermore, these numbers do not account for the emotional costs to patients and their families.

Unfortunately, to date, there are no disease-modifying medications. The presumption of failed clinical trial studies is that intervention may be too late in the course of the disease [1]. Thus, current research on AD includes significant effort to identify biomarkers decades before the threshold for clinical diagnosis is met [2]. Most current biomarker studies focus on measures of β amyloid (Aβ) detected by positron emission tomography (PET) imaging or measured from cerebrospinal fluid [3], [4], [5]. However, nearly 75% of cognitively normal individuals aged 70–79 years are free of significant amyloid on PET imaging [6]. Furthermore, studies of younger individuals show differences in brain structure and cognitive ability related to *APOE* genotype status [7] or vascular risk factors [8] at a time when pathologic and in vivo measures of AD pathology are virtually absent. These data suggest that factors other than cerebral amyloidosis during early life may result in increased risk for later-life dementia.

There is mounting evidence suggesting a more variable profile in which indices of the neurodegenerative processes (as indicated by changes in cognitive and brain structure) may precede amyloidosis [9]. Despite results that challenge the Aβ model for AD pathogenesis, most clinical trial studies remain centered around amyloid as the primary target for drug development [10], [11]. We propose that the reason clinical trial studies have largely failed is because the pharmaceutical and AD research communities assume AD is a single disease. In fact, it has been well documented that sporadic AD is a heterogeneous disease [12]. Decades ago, the variability of the AD profile and its implication were more widely considered [13], [14], [15], but once the NINCDS-ADRDA diagnostic criteria was put into widespread practice, it was largely abandoned. This may be because the definite diagnosis of dementia depends on autopsy data, requiring widespread distribution of amyloid plaques and neurofibrillary tangles for diagnosis [16]. Neuropathologic studies, however, suggest that other pathologies often co-occur and at times may lead to antemortem diagnosis of AD, even when neuropathologic criteria are not met [12].

Consider now cancer as a model for rethinking the treatment strategy for AD. Like AD, cancer at its end stages is widespread and etiology is difficult to determine. Today, the diagnosis of cancer itself is uninformative. Rather research has determined that there are over 100 different types of cancers, identified in part by symptom variability. As a result of recognizing multiple forms of the disease, drug treatments target each type. Currently, a cancer diagnosis requires additional testing to pinpoint subtype and stage. Depending on the answer, different treatment options are considered. For the diagnosis of leukemia alone, there are over 70 treatment options.

For AD, many different drug discovery studies are ongoing but there is no overt strategy of identifying the “types of AD” as potential targets for different drug treatment. Our analogy to cancer is not a clean one. Cancer is a different disease in which biopsy can differentiate between cancer cells and normal ones. The current work on AD biomarkers, however, is an equivalent attempt to find the earliest indicator of disease, similar to detecting preclinical cancer cells.

Through biomarker research, the evidence suggests that there are people who are symptomatic for AD that have amyloid and those who do not. Furthermore, there are people without cognitive impairment who have amyloid and those who do not. On commonly used assessment methods, cognitive impairment profiles include those that are predominantly amnestic and those that are not. In fact, for almost every positive study in favor of a particular AD risk factor or biomarker, there are contradictory negative findings.

We submit that the primary question we should collectively seek to answer is whether the path to effective AD treatment lies in identifying ADs, potentially resulting in multiple drug targets rather than one. It is likely that the data needed to support this hypothesis have already been generated. A shift from the presumption of a single disease to multiple diseases would reframe the drug development strategy for AD. Although there have been and continue to be clinical trial studies of unique drug compounds, they all are based on the same premise of seeking a cure for a single disease which has been well documented as heterogeneous in nature. Current research has presumed that the failure of past studies stems from intervention that has occurred too late in the disease process and thus much money and effort is being spent on trying to target the disease at its earliest stages. Doing so has resulted in trying to identify people at risk for disease based on specific clinical profiles, including still uncertain expensive biomarkers that require procedures that can be invasive and thus lead to inherently biased study samples. The difference between what we are proposing and what is currently being done is we contend that AD is not a heterogeneous disease, it is multiple diseases, and each type is expressing itself, even at the asymptomatic stages, through these varying clinical profiles. Once each type is identified, the opportunity exists for finding its unique drug target.

In summary, the focus of future Alzheimer's research should be on:

1. Identifying AD subpopulations
2. Understanding the structure of each population based on the molecular data
3. Building stratification model(s) (for disease subtypes, drug response, and prognosis)
4. Identifying new therapeutic targets

## Closing remarks

Expression-based classification of patients into groups of interest has been a challenge due to patient heterogeneity. One potential solution to overcome some of these challenges has been to use prior knowledge in the form of information around known signaling pathways or protein-protein interaction networks into the statistical analysis procedure, resulting in a more integrated approach. The activity of these entire pathways or networks is then used, rather than the expression levels of single genes, to stratify patients into their respective groups. It has been shown in a number of studies that these approaches based on utilization of prior knowledge achieve better results (e.g. accuracy, reproducibility and robustness of the models) than purely data-driven approaches for such applications as biomarker identification, patient stratification [17], [18], and clustering analysis for subgroup identification [19]. Moreover, these approaches subsequently allow for a wide spectrum of unique applications, such as mechanism reconstruction and drug target identification [20].

Four decades after the United States declared a “war” on cancer, there have been battles won and plenty of advances but no final victory. The medical field has spent far fewer years with a similar focus on Alzheimer's; it was only 2 years ago the Obama administration unveiled a national plan to find a cure by 2025. We, the authors, think that by asking the question of finding a cure for a disease, rather than multiple diseases, we are impeding our ability to understand what the data we already have on hand are trying to tell us. We could well be on the brink of “controlling” AD but to push us to the other side of increasing treatment success, we need to re-evaluate what we are trying to target. We further posit that the likely best cure for AD is in its prevention. As we gain success in sharpening our understanding of the biomarkers for each type of disease at its earliest stages, we will also enhance identification of effective prevention strategies. Before there is a cure or as we have argued, cures, society needs to promote interventional strategies to lessen the disease's impact on those already showing risk for disease. The alternative is finding ourselves on an ever-widening abyss, into which many of our loved ones, friends, and colleagues are likely to fall.
